# To what extent can behaviour change techniques be identified within an adaptable implementation package for primary care? A prospective directed content analysis

**DOI:** 10.1186/s13012-017-0704-7

**Published:** 2018-02-17

**Authors:** Liz Glidewell, Thomas A. Willis, Duncan Petty, Rebecca Lawton, Rosemary R. C. McEachan, Emma Ingleson, Peter Heudtlass, Andrew Davies, Tony Jamieson, Cheryl Hunter, Suzanne Hartley, Kara Gray-Burrows, Susan Clamp, Paul Carder, Sarah Alderson, Amanda J. Farrin, Robbie Foy, on behalf of the ASPIRE programme team, Vicky Ward, Robert West, Martin Rathfelder, Claire Hulme, Judith Richardson, Tim Stokes, Ian Watt

**Affiliations:** 1 Leeds Institute of Health Sciences, University of Leeds, Leeds, UK; 2 School of Psychology, University of Leeds, Leeds, UK; 3 Bradford Institute for Health Research, Bradford Royal Infirmary, Bradford, UK; 4 Leeds Institute of Clinical Trials Research, University of Leeds, Leeds, UK; 5 Yorkshire and Humber Academic Health Science Network, Wakefield, UK; 6 School of Dentistry, University of Leeds, Leeds, UK; 7 West Yorkshire Research and Development, Bradford Districts Clinical Commissioning Group, Bradford, UK

**Keywords:** Implementation intervention, Behaviour change techniques, Theoretical Domains Framework, Discriminant content analysis, Audit and feedback, Educational outreach, Computerised prompts, Clinical reminders

## Abstract

**Background:**

Interpreting evaluations of complex interventions can be difficult without sufficient description of key intervention content. We aimed to develop an implementation package for primary care which could be delivered using typically available resources and could be adapted to target determinants of behaviour for each of four quality indicators: diabetes control, blood pressure control, anticoagulation for atrial fibrillation and risky prescribing. We describe the development and prospective verification of behaviour change techniques (BCTs) embedded within the adaptable implementation packages.

**Methods:**

We used an over-lapping multi-staged process. We identified evidence-based, candidate delivery mechanisms—mainly audit and feedback, educational outreach and computerised prompts and reminders. We drew upon interviews with primary care professionals using the Theoretical Domains Framework to explore likely determinants of adherence to quality indicators. We linked determinants to candidate BCTs. With input from stakeholder panels, we prioritised likely determinants and intervention content prior to piloting the implementation packages. Our content analysis assessed the extent to which embedded BCTs could be identified within the packages and compared them across the delivery mechanisms and four quality indicators.

**Results:**

Each implementation package included at least 27 out of 30 potentially applicable BCTs representing 15 of 16 BCT categories. Whilst 23 BCTs were shared across all four implementation packages (e.g. BCTs relating to feedback and comparing behaviour), some BCTs were unique to certain delivery mechanisms (e.g. ‘graded tasks’ and ‘problem solving’ for educational outreach). BCTs addressing the determinants ‘environmental context’ and ‘social and professional roles’ (e.g. ‘restructuring the social and ‘physical environment’ and ‘adding objects to the environment’) were indicator specific. We found it challenging to operationalise BCTs targeting ‘environmental context’, ‘social influences’ and ‘social and professional roles’ within our chosen delivery mechanisms.

**Conclusion:**

We have demonstrated a transparent process for selecting, operationalising and verifying the BCT content in implementation packages adapted to target four quality indicators in primary care. There was considerable overlap in BCTs identified across the four indicators suggesting core BCTs can be embedded and verified within delivery mechanisms commonly available to primary care. Whilst feedback reports can include a wide range of BCTs, computerised prompts can deliver BCTs at the time of decision making, and educational outreach can allow for flexibility and individual tailoring in delivery.

**Electronic supplementary material:**

The online version of this article (10.1186/s13012-017-0704-7) contains supplementary material, which is available to authorized users.

## Background

Dissemination of best practice, usually via clinical guidelines, is rarely sufficient by itself to implement effective or de-implement ineffective or harmful treatments [1, 2]. Observed variations in adherence to clinical recommendations are often poorly explained by routinely available patient or practice variables and are likely to be attributable to differences in clinical or organisational behaviour [3, 4]. There is generally a need for active implementation strategies to translate evidence into routine care [5]. Implementation strategies have important but variable effects (absolute effect sizes range from 3 to 16% [1, 6–11]). Research resources are potentially wasted on randomised trials of implementation strategies that do not advance understanding of what makes such strategies likely to be more or less effective [12–15]. Attempts to improve this understanding are often hampered by insufficient description of intervention content [10, 11, 16, 17]. Accurate descriptions are needed to interpret heterogeneity of effects within systematic reviews, inform replication of promising features of implementation strategies and guide the exclusion of less-effective features.

Many implementation studies focus on one clinical behaviour or condition [18, 19]; it is uncertain whether an implementation strategy developed for one problem will work for another. This is particularly problematic for settings such as primary care where clinicians need to manage a wide variety of conditions. It is impracticable and inefficient to develop and evaluate an implementation strategy for every clinical guideline. Adaptable strategies are required, which can potentially be generalised across different quality indicators and sustainably integrated within existing resources.

Targeting implementation strategies according to determinants of adherence (also known as barriers, enablers, or facilitators that influence or affect behaviour) may improve their effectiveness [16, 20, 21]. The Theoretical Domains Framework (TDF) offers a structured approach for exploring the perceptions of those whom are targets for the intervention [12, 22–24], whilst the Behaviour Change Taxonomy outlines 16 categories of 93 specific, theoretically informed or evidence-based behaviour change techniques (BCTs) that are hypothesised to change behaviour [23, 25]. BCTs are observable, replicable and irreducible ‘active ingredients’ that offer a common language with which to describe intervention content. There is a lack of guidance on how best to operationalise, tailor content and combine BCTs to enhance effectiveness. For example, motivation may be increased by fear of negative consequences, but excessive fear may inhibit action [26].

We previously screened the National Institute for Health and Care Excellence (NICE) guidelines and associated quality standards to identify 2365 guideline recommendations considered relevant to UK primary care [27]. Following a cross-sectional analysis of patient data [4], we derived four ‘high-impact’ quality indicators based on criteria including as follows: the burden of illness, the potential for significant patient benefit from improved practice, the likelihood of cost savings without patient harm and the feasibility of measuring change using routinely collected data. The four quality indicators comprised the following: diabetes control (achieving all recommended target levels of haemoglobin A1c (HbA1c), blood pressure (BP) and cholesterol in patients with type 2 diabetes); risky prescribing, largely focusing on avoiding adverse gastrointestinal, renal and cardiac effects of non-steroidal anti-inflammatory drugs (NSAIDs) and anti-platelet drugs; BP control in patients at high cardiovascular disease risk; and anticoagulation for stroke prevention in patients with atrial fibrillation (AF) [28].

We aimed to systematically develop an implementation package and adapt it for the four quality indicators. Whilst intervention descriptions have previously been coded for the presence of BCTs [25, 29], and the delivery of intended BCTs [30], we have not yet encountered any published studies prospectively evaluating intervention content before evaluation. Prospective coding reduces the likelihood of post hoc rationalisation whereby intervention descriptions are influenced by knowledge of evaluation outcomes [31]. In this paper, we will address two research questions. First, were the BCTs we intended to operationalise identified by an external coder during a directed content analysis? Second, which verified BCTs were shared or unique to the implementation package adapted for four quality indicators?

## Methods

**Fig. 1:**
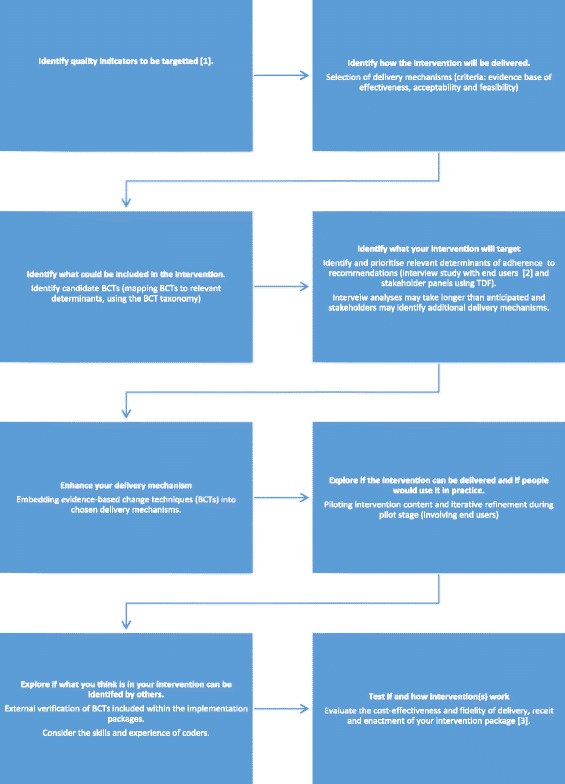
Multi-staged approach to develop and content analyse BCT content of implementation package. Multi-staged approach to develop and content analyse an implementation package with embedded BCTs adapted for four quality indicators

We used an overlapping multi-staged approach adopting an interpretivist stance to design and verify the content of an implementation package adaptable for four quality indicators using the BCT taxonomy (Fig. 1 & Table 1). All research was undertaken in West Yorkshire, England, which covers a socioeconomically diverse population of 2.2 million residents [32]. Approximately 300 general practices are organised within 10 clinical commissioning groups (CCGs). Demographically, they are broadly typical of the national average, with the exception of higher deprivation levels [33].

- *Stage 1: selecting delivery mechanisms*. We aimed to create an adaptable implementation package based upon resources typically available within primary care. Commonly used delivery mechanisms known to be effective [1] were selected by the intervention development team: audit and feedback [8], educational outreach [11] and computerised prompts and reminders [10]. We aimed to embed evidence-based features known to increase their impact, e.g. repeated feedback of audit data, the requirement for users to select a reason for over-riding a computerised prompt [8, 10].
- *Stage 2: identifying candidate BCTs*. We aimed to enhance selected delivery mechanism effects by embedding BCTs (e.g. ‘feedback on behaviour’ or ‘action planning’ [23]). Team members with experience of applying behavioural theories to implementation strategies (LG, RL, RM and RF) independently mapped the 12 determinants from the TDF [34] to one or more of the 16 broad BCT categories and then to individual BCTs using an electronic spreadsheet. Results were collated and any BCT category nominated by three or more researchers was considered eligible. The team discussed discrepancies until consensus was agreed. We aimed to generate an inclusive list of ‘candidate’ change techniques. A matrix was produced to indicate BCTs with the potential to target one or more theoretical determinants.
- *Stage 3: identifying and prioritising relevant theoretical determinants of behaviour*. We have earlier described the methods and findings of interviews with primary care clinicians and managers to explore determinants of adherence [35]. Given the timeframe required to analyse and compare four substantial sets of interview data, emerging interview findings (themes and illustrative quotes) based on interviewer field notes and exploration of frequency data were compiled for each indicator. We convened a series of multi-disciplinary stakeholder panel meetings, one for each quality indicator. The intervention development team used their previous knowledge to identify stakeholders involved in achieving each quality indicator. We invited five to ten stakeholders representing clinicians (general practitioners, practice nurses, pharmacists), practice managers, quality improvement specialists and service commissioners. All invited stakeholders were willing to participate in the consensus process. We presented them with emerging interview analyses [35] (frequency data and illustrative quotes for each determinant of achievement). After reviewing the range of determinants, stakeholders were asked to suggest additional professional or organisational determinants and contextualise our findings. Candidate BCTs (identified during the mapping exercise in stage 2) and messages which could be framed were reviewed for potential fit within the organisational context of primary care and feasibility of operationalisation within the different delivery mechanisms. Evidence-based intervention features were discussed to explore their acceptability prior to implementation. Field notes were used to record discussion points. We simultaneously convened a parallel group of nine patient and public representatives and followed similar methods. The research team communicated key messages from one panel to another. Suggestions from both groups were reviewed by the intervention development team, including social scientists and clinicians, to maximise acceptability and feasibility.

**Table 1:** Standards for Reporting Qualitative Research (SRQR)

	Title and abstract	
S1	Title	Indicate qualitative approach ‘A prospective directed content analysis’ p1.
S2	Abstract	Abstract includes background, purpose, methods, results and conclusions p2/3.
	Introduction	
S3	Problem formulation	Significance of the problem studied p3, relevant theory p4, and empirical work p3/4.
S4	Purpose or research question	Specific research objectives and research questions p4/5.
	Methods	
S5	Qualitative approach and research paradigm	Multi-method qualitative approach (individual semi-structured interviews, observation, consensus panel work and a directed content analysis) informed by psychological theory p5, adopting an interpretivist stance p5.
S6	Researcher characteristics and reflexivity	Researcher personal attributes, qualifications/experience p5& 7, and relationship with participants’ p7.
S7	Context	Setting and salient contextual features p5.
S8	Sampling strategy	How and why research participants’ p7 and p8 selected and rationale for no further sampling p8.
S9	Ethical issues pertaining to human subjects	Review p7, consent p7 and data security issues N/A.
S10	Data collection methods	Types of data collected p7/8, data collection procedures (start/stop dates, analysis plan and any modifications p7/8).
S11	Data collection instruments and technologies	Instruments (guides/questionnaires N/A opportunistic conversations) and devices (audio recorders N/A field notes taken).
S12	Units of study	Number and relevant characteristics of participants p7/8, documents N/A or events N/A.
S13	Data processing	Methods prior to and during analysis (transcription N/A, data entry N/A, data management (see methods for different stages).
S14	Data analysis	Process inferences, themes identified and developed (reported in separate paper), who involved p7/8.
S15	Techniques to enhance trustworthiness	Rationale for member checking (not done), audit trail p7/8, triangulation N/A.
	Results/Findings	
S16	Synthesis and interpretation	Main findings and integration with prior research or theory p10-end.
S17	Links to empirical data	Evidence to substantiate analytic findings Tables 1, 2, 3, and 4
	Discussion	
S18	Integration with prior work, implications, transferability and contribution(s) to the field	Main findings p8, how they challenge, support or elaborate on earlier scholarship p10-end. Scope of application/generalizability p10. Identification of unique contribution to scholarship p10-end.
S19	Limitations	Trustworthiness and limitations p11 of findings.
	Other	
S20	Conflicts of interest	Perceived influences and how managed p16.
S21	Funding	Source of funding and role of funders in data collection, interpretation and reporting p16.

Following the stakeholder panels, we further analysed interview findings using the TDF [34, 36] to identify the most prominent determinants and high-level themes. Determinants were grouped into four categories: core, prominent, less evident and not identified. Determinants considered *core* to all four quality indicators (i.e. consistently raised regardless of quality indicator) were ‘social and professional role’ and ‘environmental context and resources’; those considered *prominent* (i.e. determinants which varied in importance) were ‘beliefs about consequences’, ‘social influences’, ‘knowledge’ and ‘memory, attention and decision processes’; and those considered *less evident* were ‘skills’, ‘beliefs about capabilities’ and ‘motivation and goals’. Determinants ‘emotion’ and ‘behavioural regulation’ were *not identified*. These data informed the content of follow-up feedback reports.

- *Stage 4: designing intervention content*. We created a prototype outline for each delivery mechanism (feedback report, educational outreach session and computerised prompts and reminders) including features known to enhance effectiveness [10, 16] and findings from stages 1 to 3. Computerised prompts were not developed for diabetes or blood pressure control because they were already widely used. Stakeholders also suggested patient-directed checklists to guide discussions around diabetes and blood pressure control respectively. We then embedded candidate BCTs (stage 2) that could target modifiable determinants of adherence (stage 3). Using contextual data from interviews, the prototype was adapted and tailored for each quality indicator. We used the vocabulary and experiences expressed in interviews with health care professionals and stakeholder panellists to tailor BCT content within delivery mechanisms. A graphic designer enhanced the final intervention template. All prototypes were reviewed by the intervention development group for feasibility and acceptability prior to piloting.
- *Stage 5: piloting intervention content and refinement*. We piloted each delivery mechanism for all quality indicators with five consenting general practices involved in our earlier interview study [35]. Brief opportunistic semi-structured interviews were conducted by EI a social scientist researcher, previously unknown to the practice. She directly observed the delivery of each educational outreach session. Feedback reports, patient-directed checklists and protocols for computerised prompts were presented as written documents. She conducted brief, opportunistic semi-structured interviews with relevant practice staff (six GPs, two practice managers and three practice nurses). Participants commented on the acceptability and feasibility of prototype delivery mechanisms. Field notes were taken and reviewed by the intervention development team, leading to refinements of the first feedback report template. Participants were not asked to comment on the presence or absence of BCTs. The impact of BCTs on hypothesised determinants was not explored when piloting interventions.
- *Stage 6: verification of BCTs included within implementation packages.* Three trained coders (LG, RL and KG-B) applied the BCT Taxonomy version 1 in two stages to identify intended and verified BCTs [23]. First, two members of the intervention development team (LG and RL) assessed which BCTs were included in a sample of delivery mechanisms (feedback reports, educational outreach visit plan for one quality indicator) to identify intended content. A directed content analysis exploring the consistency of identification was completed by a researcher external to the intervention development team (KG-B) [37]. All implementation package delivery mechanisms were coded to one or more BCTs independently by LG and KG-B. Discrepancies were noted and decisions made about sufficient evidence to verify inclusion. We then examined the extent to which BCT content was shared across (or unique to) the adapted implementation package and different delivery mechanisms.

### Ethics

The ASPIRE programme was reviewed by the Leeds Central Research Ethics Committee in June 2012 (12/YH/0254) and National Health Service research governance permissions granted by the West Yorkshire Commissioning Support Unit.

## Results

BCTs with the potential to target one or more theoretical determinants (Table 2) were identified in a matrix (Table 3). We identified 30 BCTs with potential to target determinants from our emerging interview study findings and stakeholder panellists (Additional file 1: Table S1 and Table 4). BCTs that could not be operationalised within our delivery mechanisms or fit within the context of primary care resources were considered ineligible (Table 5).

**Table 2:** Perceived determinants of adherence prioritised for intervention development

Layered identification of theoretical determinants	Capability				Opportunity		Motivation				Other
	Physical	Psychological			Social	Physical	Reflective			Automatic	
	Skills	Knowledge	Memory	Behavioural regulation	Social influences	Environmental context	Beliefs about capabilities	Beliefs about consequences	Social professional role	Emotion	Patient factors
1. Interviewer consensus	AF	All		BP RP DC	DC	AF BP DC	BP RP DC	BP RP	All	AF RP DC	All
2. Emerging interview finding (most frequently cited determinant)	BP	All		AF RP DC	All	AF BP DO	DC	All	All		All
3. Consensus panel of clinical and patient stakeholders	DC		DC								
4. Extended qualitative analysis of interview data		AF RP DO	AF RP	AF	BP DC	All		All	All		All
Combined analysis	AF BP DC	All	AF RP DC	All	All	All	BP RP DC	All	All	AF RP DC	All

*AF* Anticoagulation for atrial fibrillation, *BP* blood pressure, *RP* risky prescribing, *DC* diabetes control

**Table 3:** Candidate behaviour change techniques mapped to determinants of behaviour ordered by core, prominent and less-evident determinants identified during previous interview study [35]

Potential behaviour change technique (BCT) categories [23] ordered by likelihood of targeting core, prominent and less-evident determinants	Importance of determinant										
	Core to all indicators		Prominent across indicators				Less evident			Not identified	
	Environmental context	Social professional role	Knowledge	Memory	Social influences	Beliefs about consequences	Skills	Beliefs about capabilities	Motivation and goals	Emotion	Behavioural regulation
Social support	●	●			●			●		●	
Antecedents	●			●							●
Comparison of behaviour		●	●		●		●	●	●	●	
Feedback and monitoring		●		●		●		●	●		●
Identity		●								●	
Covert learning		●								●	
Comparison of outcomes			●		●	●			●		
Natural consequences			●			●			●	●	
Shaping knowledge			●				●				
Goals and planning				●				●	●		●
Repetition and substitution				●			●	●			●
Associations				●					●	●	●
Regulation				●						●	●
Reward and threat					●				●		
Self-belief								●			
Scheduled consequences									●		●
Number of potentially relevant BCT categories	2	5	4	6	4	3	3	6	8	7	7

A dot signifies a BCT with the potential to target a determinant

**Table 4:** Intervention description based on TIDIER [25]

	Audit and feedback	Educational outreach (supplemented by audit and feedback)	Computerised prompts and paper-based reminders
Rationale	We aimed to develop an adaptable implementation package which can be implemented within existing primary care systems and resources and adapted to specifically target barriers to change for four quality indicators.		
Control interventions	Both control and intervention practices will be exposed to standard practice quality improvement initiatives e.g. national guidelines and financial incentives.		
Materials and training	*Practice-specific quarterly audit reports* Each report contained a comparison of the practices’ behaviour or outcomes in relation to the other participating practices within their locality (i.e. their Clinical Commissioning Group responsible for commissioning services) and all participating practices across West Yorkshire to reflect on progress and to prompt the need for change. Information on clinical recommendations and potential change strategies were provided. Consequences of inaction were described. Practices were encouraged to set goals based on graded tasks (based on the number of clinical recommendations and number of patients to be targeted within each recommendation) and use an action planning template to detail who would do what; in what circumstances; and how and when the achievement would be reviewed. Subsequent reports included potential actions identified during outreach sessions. *Computerised searches* Searches could be included in the practice’s Clinical Information System (CIS) to systematically identify all patients whose care should be reviewed and facilitate repeat searching. *Short and longer significant event audit (SEA) templates* Short and longer forms were developed for risky prescribing and anticoagulation for AF indicators to facilitate root cause analyses and action planning from harmful events or near misses.	We commissioned for and recruited experienced Pharmacist facilitators who received 2 days training aimed to increase motivation, prompt individual and group reflection, increase confidence and intention to act. For each outreach visit, a practice-specific outreach pack was developed containing: the most recent (and all previous) audit report(s); a session outline; an action plan template that included space for noting current performance, setting a target, identifying who will do what and review date; and templates for assessing costs and benefits. We did not articulate the discrepancy or specifically request that the team did so and although it is possible that the team might do this, they might also explain the lack of achievement away in other ways and not those related to behaviours. As we can only infer that this technique was deployed we did not code for it. Training in BCT coding requires that inferences are not made.	For risky prescribing nine *computerised prompts* were developed to be triggered within the consultation and during repeat prescribing on the basis of a clinical code algorithm for age/diagnosis/drug and duration. When triggered a brief message notified that the patient was at risk and presented one sentence of evidence-based risk (e.g. ‘This patient has CKD. NSAID use accounts for an estimated 15% of all cases of acute renal failure and 36% of drug-induced cases’). A one-click justification was required (e.g. continue with risk, add medication, or stop medication). Two prompts were developed for anticoagulation for AF but could not be made available within the study timelines. *Patient-directed checklists* Checklists were developed to facilitate shared decision making for managing blood pressure and diabetes outcomes but could not be made available within the study timelines so were not included in the directed content analysis. *Paper-based reminders* in the form of laminated information sheets were created to convey key clinical information (blood pressure, risky prescribing and anticoagulation for AF). *Pens and post-it notes* were sent to all practices with a topic specific reminder to prompt behaviour.
Supportive activities	None.	Pharmacist training included a one-day face-to-face meeting with intervention developers focussing on goal setting, action planning, clinical barriers, and persuasive communication. This was followed by a half day of independent study using a folder of supporting documentation relevant to each clinical priority. The first outreach meeting of each facilitator was observed by an experienced facilitator and feedback was given.	None.
Intervention provider	Reports, searches and templates were created by the research team.	Professional outreach education company.	Reminders were created.
Mode of delivery	Reports were sent by post and e-mail. Practices were sent invitations to use computerised searches from a task from within their clinical information system. An email was sent from the ASPIRE team to the practice manager and colleagues introducing SEA templates.	Face-to-face sessions were offered to practices.	Practices were sent invitations to use computerised prompts from a task within their clinical information system. An email was also sent from the ASPIRE team to the practice manager and colleagues alerting them to option to accept the prompts into their CIS.
Schedule and intensity	Quarterly feedback reports. Practices were offered access to searches and SEA templates at the beginning of the study and reminded of their availability via quarterly feedback reports.	Practices were offered an initial 30-min session. All practice staff involved in identifying/reviewing appropriate patients were invited to attend. A key clinical contact was identified to support practice engagement. Initial visits focussed on practice achievement data (from audit reports), identifying models of good practice, addressing barriers to change and creating an action plan to facilitate and review the change. Two days of pharmacist provision was offered to support patient identification and review. An additional follow-up visit was offered to review action plan progress and supportthe practice to create more challenging or attainable plans.	Practices were offered access to prompts at the beginning of the study and reminded of their availability via quarterly feedback reports. Practices were offered access to checklists at the beginning of the study and reminded of their availability via quarterly feedback reports. Post-it notes and pens were sent to all practices.
Tailoring	Searches could be tailored by practices, allowing them to identify patients relevant to all or individual recommendations, or adjust target values to select specific groups of patients.	Session content could be modified to practice requirements.	Prompts could be copied and modified to practice requirements.
Modifications	None.		
Fidelity of delivery, receipt and enactment	Will be assessed in the subsequent process evaluation.

**Table 5:** Behaviour change techniques excluded from intervention development or intended but not subsequently identified during content analyses

Behaviour change techniques (BCTs) for changing determinants of behaviour [35]		BCTs excluded because of delivery mechanism or contextual constraints (BCT taxonomy code reference [23])	BCTs intended but not subsequently identified by independent coder
Relevant determinants			
Core determinants ‘environmental context’ and ‘social and professional role’.	Social support	Social support emotional (3.3)	
	Antecedents	Avoidance/reducing exposure to cues for the behaviour (12.3) Distraction (12.4) Body changes (12.6)	
	Comparison of behaviour	Demonstration of the behaviour (6.1)	
	Feedback and monitoring	Monitoring of behaviour by others without feedback (2.1) Monitoring of outcomes of behaviour without feedback (2.5) Biofeedback (2.6)	
	Identity	Incompatible beliefs (13.3) Valued self-identity (13.4) Identity associated with changed behaviour (13.5)	Identification of self as role model (13.1)
	Covert learning	Imaginary punishment (16.1) Imaginary reward (16.2)	
Prominent determinants ‘knowledge’, ‘memory’, ‘social influences’ and ‘beliefs about consequences’.	Comparison of outcomes	Comparative imagining of future outcomes (9.3)	
	Natural consequences	Monitoring of emotional consequences (5.4) Information about emotional consequences (5.6)	Anticipated regret (5.5)
	Shaping knowledge	Behavioural experiments (4.4)	
	Goals and planning		Discrepancy between current behaviour and goal (1.6)
	Repetition and substitution	Behavioural practice/rehearsal (8.1) Behaviour substitution (8.2) Habit reversal (8.4) Overcorrection (8.5) Generalisation of target behaviour (8.6)	
	Associations	Cue signalling reward (7.2) Reduce prompts/cues (7.3) Remove access to the reward (7.4) Remove aversive stimulus (7.5) Satiation (7.6) Exposure(7.7) Associative learning (7.8)	
	Regulation	Pharmacological support (11.1) Reduce negative emotions (11.2) Paradoxical instructions (11.4)	
	Reward and threat	Material incentive (behaviour) (10.1) Material reward (behaviour) (10.2) Non-specific reward (10.3) Social incentive (10.5) Non-specific incentive (10.6) Self-incentive (10.7) Incentive (outcome) (10.8) Self-reward (10.9) Reward (outcome) (10.10) Future punishment (10.11)	
Less-evident determinants ‘self-belief’ and ‘scheduled consequences’	Self-belief	Mental rehearsal of successful performance (15.2) Self-talk (15.4)	Verbal persuasion about capability (15.1)
	Scheduled consequences	Behavioural cost (14.1) Punishment (14.2) Remove reward (14.3) Reward approximation (14.4) Rewarding completion (14.5) Situation-specific reward (14.6) Reward incompatible behaviour (14.7) Reward alternative behaviour (14.8) Reduce reward frequency (14.9) Remove punishment (14.10)	

### The implementation package adapted for four quality indicators

We tailored delivery mechanisms for each quality indicator: audit and feedback, educational outreach and computerised prompts and reminders. Full operational details of how each delivery mechanism was developed and intended to be delivered have been specified following TIDieR guidance [38] (Table 3). *Audit and feedback* comprised quarterly reports, computerised searches to identify patients and significant event audit templates to support root cause analyses (risky prescribing; anticoagulation for AF). Report content was designed to inform and prompt memory of clinical targets, highlight consequences of not changing behaviour, suggest potential strategies for change, give feedback on outcomes/behaviour, compare adherence with others, encourage goal setting and use of an action plan template and encourage reflection on progress towards goals. *Educational outreach* comprised an initial 30-min session to focus on action planning, increase motivation, discuss barriers to action, facilitate group reflection and increase confidence to act. Sessions were facilitated by pharmacists following 2 days training. A follow-up session was available to review goal achievement and create more challenging or attainable plans. Two days of pharmacist support were offered to support patient identification and review. *Computerised prompts and reminders* were created to reinforce clinical messages of who and what to target for each indicator. Nine computerised prompts for risky prescribing and two for anticoagulation for AF requiring a one-click justification (e.g. continue with risk, add medication, or stop medication) were developed. To avoid duplication with existing quality improvement systems, we did not develop computerised prompts for diabetes control or BP control [39]. Laminated reminders were created to convey key clinical information for BP control, anticoagulation for AF and risky prescribing. Patient-directed checklists to facilitate shared decision making were developed for distribution by practice staff in BP control and diabetes control practices.

### Identification of BCTs included within adapted implementation packages

The directed content analysis identified the BCTs embedded within the adapted implementation packages for four quality indicators. Each implementation package included at least 27 out of 30 potentially applicable BCTs (Additional file 1: Table S1), representing 15 of 16 BCT categories across the range of intervention delivery mechanisms. Each package contained multiple unique instances of the different BCTs. Four BCTs that were intended for inclusion (‘identification of self as a role model’ and ‘verbal persuasion about capability’ in educational outreach, ‘discrepancy between current behaviour and goal’ in feedback reports, and ‘anticipated regret’ in feedback reports and educational outreach) could not be verified.

### Extent of shared and unique BCT content across adapted implementation packages and delivery mechanisms

Adapted implementation packages for the different quality indicators shared common and unique BCT content. Twenty-three BCTs were shared across all four quality indicators (Additional file 1: Table S1). Twenty-seven BCTs were identified in strategies targeting risky prescribing and BP control, and 30 targeting anticoagulation for AF and diabetes control. Seven BCTs were unique to implementation packages largely focused on changing processes of care (risky prescribing and anticoagulation for AF contained BCTs relating to ‘goal setting for behaviour’ and ‘monitoring of behaviour’) and five BCTs were unique to packages targeting patient outcomes (BP control and diabetes control contained BCTs relating to ‘goal setting for outcomes’ and ‘monitoring for outcomes’). We did not operationalise ‘goal setting for behaviour’ or ‘monitoring of behaviours’ for the quality indicators relating to BP and diabetes control that focussed on outcomes of behaviour. This was only identified during the verification exercise once interventions had been delivered to participants.

It was possible to operationalise and identify intended BCTs within audit and feedback more frequently than for the other delivery mechanisms. Feedback reports for diabetes control and anticoagulation for AF contained 29 BCTs, compared to 28 for BP control and 26 for risky prescribing. Diabetes reports contained BCTs relating to ‘habit formation’, ‘reviewing outcome goals’ and suggestions for ‘restructuring the social environment’. Educational outreach for BP control and anticoagulation for AF contained 17 BCTs. Diabetes control and risky prescribing outreach contained 16 BCTs. ‘Goal setting outcome’ was not operationalised for risky prescribing nor anticoagulation for AF. ‘Information about social/environmental consequences’ was not identified for diabetes control. ‘Behavioural contract’ and ‘commitment’ were only designed to be included in significant event audit templates developed for risky prescribing and anticoagulation for AF and no other delivery mechanism. Computerised prompts were limited in the number of BCTs they could include because of their brevity but included additional BCTs not included in other mechanisms, (‘prompts/cues’, ‘pros/cons’, ‘adding objects to the environment’, ‘conserve mental resources’ and ‘credible source’). Paper-based reminders included ‘prompts and cues’ and ‘conserving mental resources’.

Our extended analysis of interview findings using the TDF identified ‘environmental context’ and ‘social influences’ as core determinants across all indicators. Antecedent BCTs to target these determinants were particularly challenging to include in our chosen delivery mechanisms (i.e. we prospectively excluded the BCTs ‘distraction’, ‘body changes’ and ‘avoidance’). ‘Adding objects to the environment’ was only operationalised in the computerised prompts developed for risky prescribing and anticoagulation for AF. Advice on ‘restructuring the physical environment’ and ‘restructuring the social environment’ were only included in BP control and diabetes control feedback reports respectively.

## Discussion

We have provided a detailed description of the core BCTs that can be identified across an adaptable implementation package and those unique to a range of delivery mechanisms commonly available to the UK primary care setting. Whilst published intervention descriptions have previously been reliably coded for the presence of BCTs [25, 29] and the delivery of intended BCTs [30], we believe that intervention content has not previously been coded prospectively by someone external to the development team prior to evaluation.

In our prospective assessment of the extent to which BCTs were verified, we identified a large proportion of shared BCTs (at least 23 of 30 eligible BCTs) representing 15 of 16 BCT categories, suggesting that prioritised BCTs can be embedded and identified across delivery mechanisms adapted for different quality indicators. Whilst educational outreach appeared to contain fewer BCTs, it was designed to be co-delivered with feedback reports. It was possible to include a greater range of BCTs in educational outreach because of the face-to-face delivery of outreach (e.g. ‘graded tasks’) and the co-delivery of audit and feedback reports. We did not verify if BCTs targeted intended determinants of behaviour nor could we verify the presence of four BCTs, implying their operationalisation was unsuccessful or that these BCTS are less visible.

Previous research retrospectively identifying BCTs within a systematic review of interventions for diabetes care targeting healthcare professionals [40] identified seven BCTs. We identified a broader range of included BCTs, at least 27 in each of our four adapted implementation packages, incorporating many of those not identified in the systematic review of diabetes interventions. This could be because we used a prospective identification process, or because we attempted to target a wider range of behavioural determinants identified from our interview and stakeholder panel findings.

### Limitations

We had to make trade-offs between what is theoretically desirable, clinically acceptable and operationally feasible within the context of delivery mechanisms and primary care resources. These trade-offs are partly reflected in our main study limitations.

Firstly, there were limitations in how we assessed and prioritised determinants of behaviour and subsequently linked them to BCTs. We acknowledge that perceptions of determinants are not necessarily predictors of behaviour [41]. We did not quantify the importance of each belief within determinants nor the relative importance of each determinant to the target population [42]. Therefore, it was not possible to identify individual psychological theories and use their corresponding evidence-based measures and instruments to develop each implementation package [43, 44]. We used emerging and extended interview findings to inform intervention development. It was not possible within our research timelines to use the extended findings to inform adaptation of educational outreach or initial feedback reports. We may not have adequately operationalised BCTs to target *core* and *prominent* determinants (‘social and professional role’ and ‘environmental context and resources’) within the following categories: ‘social support’, ‘antecedents’, ‘identity’ and ‘covert learning’ to target the determinants.

Secondly, BCTs from social cognition models and the TDF more generally focus on individual cognitions and may be insufficient to adequately target team, patient or organisational determinants.

Thirdly, although we aimed to develop an implementation package which could be adapted to target four (and potentially other) quality indicators, whether or not it is consistently effective is an empirical question. Whilst determinants of practice may only be relevant to countries with a comparable primary care organisation, methods to identify candidate BCTs and verify their presence are transferable.

Fourthly, despite the fact that trained and experienced coders conducted the directed content analyses, they may have omitted less-visible BCTs or over-coded BCT content. To minimise bias, a coder external to the intervention team conducted the directed content analysis. Whilst a single researcher performed the prospective content analysis, they had undertaken the BCT Taxonomy online training and a number of quality assurance steps (e.g. staged review of documents, meeting to discuss discrepancies). As coding was only conducted by two coders at each stage, it was considered inappropriate to statistically assess the reliability of this exercise, and more informative to identify discrepancies and discuss why these occurred and what could be done in subsequent research to improve the operationalisation of these techniques during intervention development. Coders will vary in the knowledge, skills and experience that they bring and may vary in their judgements of the presence/absence of BCTs.

Fifthly, the independent coder could not identify four BCTs that we intended to embed: ‘identification of self as role model’, ‘imaginary punishment’, ‘anticipated regret’ and ‘discrepancy between current behaviour and goal’). Coding discrepancies suggested that there is scope to either improve the operationalisation of some BCTs or the training provided prior to coding for BCTs.

Lastly, because of our research timelines, it was not feasible to draw out the high-level themes from the interview analyses or to modify interventions to address unsuccessful operationalisation as interventions were coded following dissemination of the fifth and final audit report. Adapting intervention content to include evidence-based findings in multiple domains (e.g. behavioural science and features known to enhance the effect size of implementation interventions) is challenging to deliver within research budgets and timescales. Future work could benefit from building in more time for iterative cycles to refine interventions and assessing the consistency of verification by coders with different skills and experience.

### Implications for research

We suggest that the methods and transparency of developing complex interventions to promote the implementation of evidence-based practice would be advanced by reporting the following: candidate BCTs considered eligible following an analysis of key behavioural determinants; BCTs targeting key determinants that could not be included within chosen delivery mechanisms, within the constraints of project resources, or implementation context; and the degree of success in operationalising and verifying intended BCTs. It is important to identify and report potentially eligible techniques that could not be verified for the following reasons. Firstly, process evaluations could explore whether interventions do not work because BCTs intended to target the most important determinants could not be operationalised. Secondly, implementation researchers can consider how prioritised determinants and salient BCTs could be operationalised within alternative delivery mechanisms. Thirdly, there may be scope to add to the existing BCT taxonomy novel BCTs that can be operationalised within different delivery mechanisms.

During our research, Cane et al. mapped the 93 BCTs to theoretical determinants [45] building on the earlier work of Michie and colleagues [34]. We identified many of the same BCTs. In addition, Cane identified two categories of BCTs that we did not, feedback and monitoring, and antecedents were mapped to knowledge, and antecedents to skills. Whilst these were not included in our intervention development work, we did include shaping knowledge, natural consequences and comparison of behaviour and outcomes to target knowledge. Shaping knowledge, comparison of behaviour and repetition and substitution BCTs were included to target skills. Michie is currently leading work to advance this line of research, including the development of an ontology for linking BCTs to theoretical determinants [46].

More research is required to establish how to best operationalise antecedent BCTs to target the determinants ‘environmental context and resources’ and ‘social influences’ (both team and patient). Whilst BCTs to target organisational determinants are available, they were harder to operationalise because they involve addressing relatively complex systems and social processes. We were aware of the importance of patient factors but prioritised our effort and resources on changing professional behaviour. We also recognised that there is a much larger body of research on changing patient behaviour (e.g. for diabetes) that we could not address within the constraints of one research programme. We did partly try to address this shortcoming by developing patient-directed checklists but could not complete these to our satisfaction within our timelines.

We do not know if there is an additive, synergistic or negative effect of operationalising multiple BCTs within a BCT category. Whilst implementation packages contained one or more instances of the BCT, we did not quantify the ‘dose’ given. Further, we only assessed the presence of the BCT and not whether the BCT effectively targeted the most salient determinants of behaviour. Previous research identifying the BCT content of diabetes implementation strategies has suggested that there is strength in harnessing the potential of BCTs to change multiple behaviours [40].

Many BCTs originate from health psychology theories developed to understand why patients do not change their behaviour; changing or not changing may result in health consequences for those individuals. The applicability of this evidence base for professionals who do not personally experience health consequences needs to be further explored. Delivery mechanisms such as audit and feedback are potentially relatively efficient to deliver if drawing upon routinely collected data but they are unable to guarantee the delivery, uptake and engagement of BCTs that require more intensive and expensive delivery mechanisms such as educational outreach (e.g. ‘graded tasks’, ‘problem solving’, ‘action planning’ and ‘commitment’) which allows for tailoring and engagement at the practice level.

The effectiveness of our implementation packages is being rigorously evaluated in a pair of cluster randomised controlled trials [28]. A parallel process evaluation will determine what was actually delivered, received and acted upon. The analysis will draw upon both Normalisation Process Theory [47, 48] and the TDF [35, 45]. The TDF was applied to help compare planned versus actual intervention content. Normalisation Process Theory was adopted given its relevance to understanding implementation processes, particularly how individuals and groups conceive of, engage with, enact and reconfigure work in response to an intervention. Our ongoing trials and process evaluations [28] of these packages will not identify BCTs that act together or independently to enhance effectiveness and this should be explored in subsequent research. We invite others to build upon and improve our methods in reporting the BCT content of implementation interventions.

## Conclusion

Implementation researchers need to identify effective and efficient means of selecting and adapting implementation strategies across a range of targeted quality indicators, rather than propose a new trial for every indicator (Table 5). We have demonstrated the specification and verification of BCT content for an adaptable implementation package targeting four quality indicators. We identified variable numbers of BCTs across the four adapted implementation packages and delivery mechanisms but would not claim that ‘more is better’; the ability to effectively target the most salient behavioural determinants is likely to be more important, although some delivery mechanisms may lend themselves better to adaptation than others. Whether or not an adapted implementation package is actually effective in targeting our four indicators, or others, is an empirical question.

## Additional file

Additional file 1: Table S1. Full description of intervention content by delivery mechanism and quality indicator (DOCX 19 kb)

## Abbreviations

AF: Atrial fibrillation
ASPIRE: Action to Support Practices Implementing Research Evidence
BCTs: Behaviour change techniques
BP: Blood pressure
HbA1c: Haemoglobin A1c
NICE: National Institute for Health and Care Excellence
NSAIDs: Non-steroidal anti-inflammatory drugs
TDF: Theoretical Domains Framework
TIDieR: Template for intervention description and replication

## References

[1] Grimshaw JM, Thomas RE, Maclennan G, Fraser C, Ramsay C, Vale L, Eccles M. Effectiveness and efficiency of guideline dissemination and implementation strategies. Health Technol Assess. 2002;8:1–247. 10.3310/hta8060 14960256

[2] Prasad V, Ioannidis JP (2014). Evidence-based de-implementation for contradicted, unproven, and aspiring healthcare practices. Implement Sci.

[3] Right Care. NHS Atlas of Variation in Healthcare. London: Public Health England; 2015.

[4] Willis TA, West R, Rushforth B, Stokes T, Glidewell L, Carder P, Faulkner S, Foy R (2017). Variations in achievement of evidence-based, high-impact quality indicators in general practice: an observational study. PLoS One.

[5] Foy R, Eccles M, Grimshaw J (2001). Why does primary care need more implementation research?. Fam Pract.

[6] Grimshaw J, Shirran L, E T, Mowatt G, Fraser C, Bero L (2001). Changing provider behaviour: an overview of systematic reviews of interventions. Med Care.

[7] Grimshaw JM, Thomas RE, MacLennan G, Fraser C, Ramsay CR, Vale L, Whitty P, Eccles MP, Matowe L, Shirran L (2005). Effectiveness and efficiency of guideline dissemination and implementation strategies. Int J Technol Assess Health Care.

[8] Ivers N, Jamtvedt G, Flottorp S, Young JM, Odgaard-Jensen J, French SD. Audit and feedback: effects on professional practice and healthcare outcomes. Cochrane Database Syst Rev. 2012;6:1–216. 10.1002/14651858.CD000259.pub3 PMC11338587 22696318

[9] Eccles MP, Steen IN, Grimshaw JM, Thomas L, McNamee P, Souter J, Wilsdon J, Matowe L, Needham G, Gilbert F (2001). Effect of audit and feedback, and reminder messages on primary-care referrals: a randomised trial. Lancet.

[10] Roshanov PS, Fernandes N, Wilczynski JM, Hemens BJ, You JJ, Handler SM, Nieuwlaat R, Souza NM, Beyene J, Van Spall HG (2013). Features of effective computerised clinical decision support systems: meta-regression of 162 randomised trials. BMJ.

[11] O’Brien M, Rogers S, Jamtvedt G, Oxman A, Odgaard-Jensen J, Kristofferson D, Forsetlund L, Bainbridge D, Freemantle N, Davis D (2008). Educational outreach visits: effects on professional practice and health care outcomes. Cochrane Libr.

[12] Michie S, Fixsen D, Grimshaw JM, Eccles MP. Specifying and reporting complex behaviour change interventions: the need for a scientific method. Implement Sci. 2009;4:40. 10.1186/1748-5908-4-40 PMC2717906 19607700

[13] Glasziou P, Meats E, Heneghan C, Shepperd S (2008). What is missing from descriptions of treatment in trials and reviews?. BMJ.

[14] Chalmers I, Bracken MB, Djulbegovic B, Garattini S, Grant J, Gülmezoglu AM, Howells DW, Ioannidis JPA, Oliver S (2014). How to increase value and reduce waste when research priorities are set. Lancet.

[15] Glasziou P, Altman DG, Bossuyt P, Boutron I, Clarke M, Julious S, Michie S, Moher D, Wager E (2014). Reducing waste from incomplete or unusable reports of biomedical research. Lancet.

[16] Ivers NM, Grimshaw JM, Jamtvedt G, Flottorp S, O'Brien MA, French SD, Young J, Odgaard-Jensen J (2014). Growing literature, stagnant science? Systematic review, meta-regression and cumulative analysis of audit and feedback interventions in health care. J Gen Intern Med.

[17] Johnson MJ, May CR (2015). Promoting professional behaviour change in healthcare: what interventions work, and why? A theory-led overview of systematic reviews. BMJ Open.

[18] Grimshaw JM, Thomas RE, MacLennan G, Fraser C, Ramsay CR, Vale L, Whitty P, Eccles MP, Matowe L, Shirran L (2004). Effectiveness and efficiency of guideline dissemination and implementation strategies. Health Technol Assess.

[19] Presseau J, Hawthorne G, Sniehotta FF, Steen N, Francis JJ, Johnston M. Improving diabetes care through examining, advising, and prescribing (IDEA): protocol for a theory-based cluster randomised controlled trial of a multiple behaviour change intervention aimed at primary healthcare professionals. Implement Sci. 2014;9:61. 10.1186/1748-5908-9-61 PMC4049486 24886606

[20] Baker R, Camosso-Stefinovic J, Gillies C, Shaw EJ, Cheater F, Flottorp S, Robertson N. Tailored interventions to overcome identified barriers to change: effects on professional practice and health care outcomes. Cochrane Database Syst Rev. 2010;(Issue 3) (Art. No.: CD005470. DOI: 10.1002/14651858.CD005470.pub2.) 10.1002/14651858.CD005470.pub2 PMC4164371 20238340

[21] Krause J, Van Lieshout J, Klomp R, Huntink E, Aakhus E, Flottorp S, Jaeger C, Steinhaeuser J, Godycki-Cwirko M, Kowalczyk A (2014). Identifying determinants of care for tailoring implementation in chronic diseases: an evaluation of different methods. Implement Sci.

[22] Eccles M, Grimshaw J, Walker A, Johnston M, Pitts N (2005). Changing the behavior of healthcare professionals: the use of theory in promoting the uptake of research findings. J Clin Epidemiol.

[23] Michie S, Richardson M, Johnston M, Abraham C, Francis J, Hardeman W, Eccles MP, Cane J, Wood CE (2013). The behavior change technique taxonomy (v1) of 93 hierarchically clustered techniques: building an international consensus for the reporting of behavior change interventions. Ann Behav Med.

[24] Davies P, Walker AE, Grimshaw JM. A systematic review of the use of theory in the design of guideline dissemination and implementation strategies and interpretation of the results of rigorous evaluations. Implement Sci. 2010;5:14. 10.1186/1748-5908-5-14 PMC2832624 20181130

[25] Hoffmann TC, Glasziou PP, Boutron I, Milne R, Perera R, Moher D, Altman DG, Barbour V, Macdonald H, Johnston M (2014). Better reporting of interventions: template for intervention description and replication (TIDieR) checklist and guide. BMJ.

[26] Gallagher KM, Updegraff JA (2012). Health message framing effects on attitudes, intentions, and behavior: a meta-analytic review. Ann Behav Med.

[27] Rushforth B, Stokes T, Andrews E, Willis TA, McEachan R, Faulkner S, Foy R (2015). Developing ‘high impact’ guideline-based quality indicators for UK primary care: a multi-stage consensus process. BMC Fam Pract.

[28] Willis TA, Hartley S, Glidewell L, Farrin AJ, Lawton R, McEachan RR, Ingleson E, Heudtlass P, Collinson M, Clamp S (2016). Action to support practices implement research evidence (ASPIRE): protocol for a cluster-randomised evaluation of adaptable implementation packages targeting 'high impact' clinical practice recommendations in general practice. Implement Sci.

[29] Abraham C, Wood CE, Johnston M, Francis J, Hardeman W, Richardson M, Michie S (2015). Reliability of identification of behavior change techniques in intervention descriptions. Ann Behav Med.

[30] McCullough AR, Ryan C, O’Neill B, Bradley JM, Elborn JS, Hughes CM (2015). Defining the content and delivery of an intervention to change AdhereNce to treatment in BonchiEctasis (CAN-BE): a qualitative approach incorporating the theoretical domains framework, behavioural change techniques and stakeholder expert panels. BMC Health Serv Res.

[31] Foy R, Sales A, Wensing M, Aarons GA, Flottorp S, Kent B, Michie S, O’Connor D, Rogers A, Sevdalis N (2015). Implementation science: a reappraisal of our journal mission and scope. Implement Sci.

[32] Population estimates for UK, England and Wales, Scotland and Northern Ireland, mid-2014. 2015. https://www.ons.gov.uk/peoplepopulationandcommunity/populationandmigration/populationestimates/datasets/populationestimatesforukenglandandwalesscotlandandnorthernireland. Accessed 26 Jan 2016.

[33] Lord PA, Willis TA, Carder P, West RM, Foy R (2016). Optimizing primary care research participation: a comparison of three recruitment methods in data-sharing studies. Fam Pract.

[34] Michie S, Johnston M, Abraham C, Lawton R, Parker D, Walker A (2005). Making psychological theory useful for implementing evidence based practice: a consensus approach. Qual Saf Health Care.

[35] Lawton R, Heyhoe J, Louch G, Ingleson E, Glidewell L, Willis TA, McEachan RRC, Foy R (2016). Using the theoretical domains framework (TDF) to understand adherence to multiple evidence-based indicators in primary care: a qualitative study. Implement Sci.

[36] Francis J, O’Connor D, Curran J (2012). Theories of behaviour change synthesised into a set of theoretical groupings: introducing a thematic series on the theoretical domains framework. Implement Sci.

[37] Hsieh HF, Shannon SE (2005). Three approaches to qualitative content analysis. Qual Health Res.

[38] Hoffman T, Glasziou PP, Boutron I, Milne R, Perera R, Moher D. Better reporting of interventions: template for intervention description and replication (TIDieR) checklist and guide. BMJ. 2014;348:g1687. 10.1136/bmj.g1687 24609605

[39] QOF business rules v23.0. http://www.pcc-cic.org.uk/article/qof-business-rules-v230. [http://www.pcc-cic.org.uk/article/qof-business-rules-v230] September 2017.

[40] Presseau J, Ivers NM, Newham JJ, Knittle K, Danko KJ, Grimshaw JM (2015). Using a behaviour change techniques taxonomy to identify active ingredients within trials of implementation interventions for diabetes care. Implement Sci.

[41] Peters G-JY (2014). A practical guide to effective behavior change: how to identify what to change in the first place. Eur Health Psychol.

[42] de Bruin M, Crutzen R, Peters G-JY (2015). Everything should be as simple as possible, but this will still be complex: a reply to various commentaries on IPEBA. Health Psychol Rev.

[43] Kok L. A practical guide to effective behavior change: how to apply theory- and evidence-based behavior change methods in an intervention. Eur Health Psychol. 2014;16(5):156–70.

[44] Prestwich A, Webb TL, Conner M (2015). Using theory to develop and test interventions to promote changes in health behaviour: evidence, issues, and recommendations. Curr Opinion in Psychology.

[45] Cane J, O'Connor D, Michie S (2012). Validation of the theoretical domains framework for use in behaviour change and implementation research. Implement Sci.

[46] Developing methodology for designing and evaluating theory-based complex interventions: an ontology for linking behaviour change techniques to theory [http://gtr.rcuk.ac.uk/projects?ref=MR%2FL011115%2F1#] August 2017.

[47] May CR, Mair FS, Dowrick CF, Finch TL. Process evaluation for complex interventions in primary care: understanding trials using the normalization process model. BMC Fam Pract. 2007;8:42. 10.1186/1471-2296-8-42 PMC1950872 17650326

[48] Murray E, Treweek S, Pope C, MacFarlane A, Ballini L, Dowrick C, Finch T, Kennedy A, Mair F, O'Donnell C (2010). Normalisation process theory: a framework for developing, evaluating and implementing complex interventions. BMC Med.

