# Supplementary material for: To what extent can behaviour change techniques be identified within an adaptable implementation package for primary care? A prospective directed content analysis
Source: Implement Sci. 2018 Feb 17;13:32. doi: 10.1186/s13012-017-0704-7 (PMC5816358; doi:10.1186/s13012-017-0704-7)
Supplement: Supplementary file 1 — Full description of intervention content by delivery mechanism and quality indicator (DOCX 19 kb) [file 13012_2017_704_MOESM1_ESM.docx]

Additional file 1 Table S1: Full description of intervention content by delivery mechanism and quality indicator

| **Determinants of behaviour** | **BCTs verified by independent coder (BCT taxonomy code reference)** | | **Implementation package (see subsequent headings for variation in delivery mechanisms by quality indicator)** | | | | **Audit and feedback** | | | | **Educational outreach** | | | | **Computerised prompts and/or paper-based reminders** | | | | **Illustrative intervention content (see Table 4 for fuller description)** |
| --- | --- | --- | --- | --- | --- | --- | --- | --- | --- | --- | --- | --- | --- | --- | --- | --- | --- | --- | --- |
|  |  |  | **Risky prescribing** | **Diabetes control** | **Anticoagulation** | **Blood pressure control** | **Risky prescribing** | **Diabetes control** | **Anticoagulation** | **Blood pressure control** | **Risky prescribing** | **Diabetes control** | **Anticoagulation** | **Blood pressure control** | **Risky prescribing** | **Anticoagulation** | **Blood pressure control** | **Diabetes control (not developed to prevent overlap with other QI initiatives)** |  |
| ‘environmental context’  ‘social and professional role’ and ‘social influences’ | Social support | Social support unspecified (3.1) | ● | ● | ● | ● | ● | ● | ● | ● | ● | ● | ● | ● |  |  |  |  | Asked to identify team members who can provide practical and emotional social support towards the achievement of behavioural and outcome goals. |
|  |  | Social support practical (3.2) | ● | ● | ● | ● | ● | ● | ● | ● | ● | ● | ● | ● |  |  |  |  |  |
| ‘environmental context’ and  ‘memory’ | Antecedents | Restructuring the physical environment (12.1) |  |  |  | ● |  |  |  | ● |  |  |  |  |  |  |  |  | Staff advised to spend time with others to share good practice. Prompts and reminders of clinical targets were added to the environment. |
|  |  | Restructuring the social environment (12.2) |  | ● |  |  |  | ● |  |  |  |  |  |  |  |  |  |  |  |
|  |  | Adding objects to the environment (12.5) |  |  |  |  |  |  |  |  |  |  |  |  | ● | ● |  |  |  |
| ‘social and professional role’, ‘knowledge’ and ‘social influences’ | Comparison of behaviour | Social comparison (6.2) | ● | ● | ● | ● | ● | ● | ● | ● | ● | ● | ● | ● |  |  |  |  | Performance data were compared to others in the Clinical Commissioning administrative group and across West Yorkshire. Information on approval/disapproval from local opinion leaders and national guidance. |
|  |  | Information about others’ approval (6.3) | ● | ● | ● | ● | ● | ● | ● | ● | ● | ● | ● | ● |  |  |  |  |  |
| ‘social and professional role’, ‘memory’ and ‘beliefs about consequences’. | Feedback and monitoring | Feedback on behaviour (2.2) | ● | ● | ● | ● | ● | ● | ● | ● |  |  |  |  |  |  |  |  | Feedback was given on processes and outcomes of behaviour. Search strategies provided for self-monitoring. |
|  |  | Self-monitoring of behaviour (2.3) | ● | ● | ● | ● | ● | ● | ● | ● |  |  |  |  |  |  |  |  |  |
|  |  | Self-monitoring of outcomes of behaviour (2.4) | ● | ● | ● | ● | ● | ● | ● | ● |  |  |  |  |  |  |  |  |  |
|  |  | Feedback on outcomes of behaviour (2.7) |  | ● |  | ● |  | ● |  | ● |  |  |  |  |  |  |  |  |  |
| ‘social and professional role’ | Identity | Framing/reframing (13.2) |  |  | ● |  |  |  | ● |  |  |  |  |  |  |  |  |  | Information framed in terms of reducing patient risk and harm. Team asked to identify role models. |
|  | Covert learning | Vicarious consequences (16.3) | ● | ● | ● | ● | ● | ● | ● | ● |  |  |  |  |  |  |  |  | Information on potential adverse events. Vicarious consequences of harms and near misses. |
| ‘knowledge’,  ‘social influences’ and ‘beliefs about consequences’ | Comparison of outcomes | Credible source (9.1) | ● | ● | ● |  | ● | ● | ● | ● | ● | ● | ● | ● | ● | ● |  |  | Communication from credible sources i.e. local clinicians. Invited to identify and compare pros and cons of changing behaviour or not, and future outcomes for the patient, practice and the health service. |
|  |  | Pros and cons (9.2) | ● | ● | ● | ● | ● |  | ● |  | ● | ● | ● | ● | ● | ● |  |  |  |
| ‘knowledge’ and ‘beliefs about consequences’ | Natural consequences | Information about health consequences (5.1) | ● | ● | ● |  | ● | ● | ● | ● | ● | ● | ● | ● | ● | ● |  |  | Information about the health, emotional, social and environmental consequences of the behaviour and the salience of these were provided. Awareness raised of future regret. |
|  |  | Salience of consequences (5.2) | ● | ● | ● | ● | ● | ● | ● | ● |  |  |  |  |  |  |  |  |  |
|  |  | Information about social/environmental consequences (5.3) | ● | ● | ● | ● | ● | ● | ● | ● | ● |  | ● | ● |  |  |  |  |  |
| ‘knowledge’ | Shaping knowledge | Instruction on how to perform the behaviour (4.1) | ● | ● | ● | ● | ● | ● | ● | ● | ● | ● | ● | ● |  |  |  |  | Advice was given on how to conduct difficult consultations. Information about situations that predict the performance of the behaviour. |
|  |  | Information about antecedents (4.2) | ● | ● | ● | ● | ● | ● | ● | ● |  |  |  |  |  |  |  |  |  |
|  |  | Re-attribution (4.3) | ● | ● | ● | ● | ● | ● | ● | ● |  |  |  |  |  |  |  |  |  |
| ‘memory’ | Goals and planning | Goal setting behaviour (1.1) | ● | ● | ● | ● | ● | ● | ● | ● | ● |  | ● |  |  |  |  |  | Attention was drawn to the discrepancy between current behaviour and goal. Problem-solving discussion to identify what is already done well and what could be done better. Asked to create an action plan that involved setting goals for behaviour and outcomes. Outcome goals reviewed. |
|  |  | Problem solving (1.2) | ● | ● | ● | ● | ● | ● | ● | ● | ● | ● | ● | ● |  |  |  |  |  |
|  |  | Goal setting outcome (1.3) |  | ● | ● | ● |  | ● | ● | ● |  | ● |  | ● |  |  |  |  |  |
|  |  | Action planning (1.4) | ● | ● | ● | ● | ● | ● | ● | ● | ● | ● | ● | ● |  |  |  |  |  |
|  |  | Review behavioural goals (1.5) | ● | ● | ● | ● | ● | ● | ● | ● | ● | ● | ● | ● |  |  |  |  |  |
|  |  | Review outcome goals (1.7) |  | ● | ● | ● |  | ● | ● | ● |  | ● | ● | ● |  |  |  |  |  |
|  |  | Behavioural contract (1.8) | ● |  | ● |  | ● |  | ● |  |  |  |  |  |  |  |  |  |  |
|  |  | Commitment (1.9) | ● |  | ● |  | ● |  | ● |  |  |  |  |  |  |  |  |  |  |
|  | Repetition and substitution | Habit formation (8.3) |  | ● |  |  |  | ● |  |  |  |  |  |  |  |  |  |  | Staff invited to rehearse what works well to increase skills. Practices asked to set increasingly difficult goals to achieve the behaviour. |
|  |  | Graded tasks (8.7) | ● | ● | ● | ● |  | ● | ● | ● | ● | ● | ● | ● |  |  |  |  |  |
|  | Associations | Prompts/cues (7.1) | ● | ● | ● | ● | ● | ● | ● | ● | ● | ● | ● | ● | ● | ● | ● |  | Computerised protocols acted as reminders during consultations. A laminate reminder acted as a prompt for BP targets for patients at high cardiovascular risk. |
|  | Regulation | Conserving mental resources (11.3) | ● | ● | ● | ● | ● | ● |  | ● |  |  |  |  | ● | ● |  |  | Advised on strategies to reduce negative emotions. Provided with mental resources to act as reminders of indicators. |
| ‘social influences’ | Reward and threat | Social reward (10.4) | ● | ● | ● | ● | ● | ● | ● | ● | ● | ● | ● | ● |  |  |  |  | Informed that behaviours may improve performance on national incentive schemes. Practices congratulated for progress and reminded of professional and legal responsibilities to prevent risk to patients. |
| ‘beliefs about capabilities’ | Self-belief | Focus on past success (15.3) | ● | ● | ● | ● | ● | ● | ● | ● |  |  |  |  |  |  |  |  | Persuasive messages about capability and past successes. |
| Total number of BCTs verified by independent coder | | | 27 | 30 | 30 | 27 | 26 | 29 | 29 | 28 | 16 | 16 | 17 | 17 | 6 | 6 | 1 |  |  |

●**=present**
